# Impact of Borna Disease Virus Infection on the Transcriptome of Differentiated Neuronal Cells and Its Modulation by Antiviral Treatment

**DOI:** 10.3390/v15040942

**Published:** 2023-04-10

**Authors:** Da Teng, Keiji Ueda, Tomoyuki Honda

**Affiliations:** 1 Division of Virology, Department of Microbiology and Immunology, Osaka University Graduate School of Medicine, Suita 565-0871, Japan; 2 Department of Virology, Okayama University Graduate School of Medicine, Dentistry and Pharmaceutical Sciences, Okayama 700-8558, Japan; 3 Department of Virology, Faculty of Medicine, Dentistry and Pharmaceutical Sciences, Okayama University, Okayama 700-8558, Japan

**Keywords:** antiviral, Borna disease virus, neuronal cells, gene expression, differentiation

## Abstract

Borna disease virus (BoDV-1) is a highly neurotropic RNA virus that causes neurobehavioral disturbances such as abnormal social activities and memory impairment. Although impairments in the neural circuits caused by BoDV-1 infection induce these disturbances, the molecular basis remains unclear. Furthermore, it is unknown whether anti-BoDV-1 treatments can attenuate BoDV-1-mediated transcriptomic changes in neuronal cells. In this study, we investigated the effects of BoDV-1 infection on neuronal differentiation and the transcriptome of differentiated neuronal cells using persistently BoDV-1-infected cells. Although BoDV-1 infection did not have a detectable effect on intracellular neuronal differentiation processes, differentiated neuronal cells exhibited transcriptomic changes in differentiation-related genes. Some of these transcriptomic changes, such as the decrease in the expression of apoptosis-related genes, were recovered by anti-BoDV-1 treatment, while alterations in the expression of other genes remained after treatment. We further demonstrated that a decrease in cell viability induced by differentiation processes in BoDV-1-infected cells can be relieved with anti-BoDV-1 treatment. This study provides fundamental information regarding transcriptomic changes after BoDV-1 infection and the treatment in neuronal cells.

## 1. Introduction

Borna disease virus (BoDV-1) is a member of *Mammalian 1 bornavirus* of the species *Orthobronavirus bornaense* in the genus *Orthobornavirus* [1,2] and a bornavirus prototype that infects cells in the central nervous system (CNS) of various animal species [3]. BoDV-1 is a non-segmented, negative-strand RNA virus that encodes at least six viral proteins: nucleoprotein (N), phosphoprotein (P), X, matrix (M), glycoprotein (G), and large protein (L) [4,5]. The BoDV-1 ribonucleoprotein complex (RNP), which consists of BoDV-1 genomic RNA (gRNA) encapsidated by the N protein and BoDV-1 polymerase complex formed by the P and L proteins, is the replication unit of the virus [4,5]. BoDV-1 produces three major protein-coding transcripts: N mRNA, X/P mRNA, and M/G/L mRNA. BoDV-1 easily establishes persistent infection in cells derived from the CNS without overt cytotoxicity.

Acute BoDV-1 infection, known as Borna disease in animals, causes fatal encephalomyelitis in horses and sheep [3]. Recently, fatal human encephalitis cases caused by bornavirus infections have been sporadically reported in Europe [6,7,8]. Besides acute infection, bornavirus establishes persistent infection in the CNS of animals, and persistently infected animals exhibit neurobehavioral disturbances resembling human psychiatric disorders [9,10,11,12]. Based on these observations, BoDV-1-infected rodents have been proposed as a model for psychiatric disorders with neurodevelopmental abnormalities, such as autism and schizophrenia [9,10,11,12]. Consequently, bornavirus infection has been investigated in human psychiatric disorders for several decades, the results of which have varied between studies. Some studies report a link between BoDV-1 infection and neuropsychiatric disorders [13], while other studies report no link between them [14]. Although several meta-analyses have suggested possible links between bornavirus infection and psychiatric disorders [15,16,17], this remains a controversial issue. As acute and persistent bornavirus infection generates CNS lesions, it is important to investigate a way to treat this viral infection. So far, interferon-α, ribavirin, T-705 (favipiravir), and a siRNA cocktail (TD-Borna) are nominated as antivirals against bornaviruses [18,19,20,21,22,23]. However, it is unclear whether bornavirus-related CNS lesions, especially functional disturbances in persistently infected neurons, can be attenuated with these treatments.

In Lewis rats perinatally infected with BoDV-1, BoDV-1 preferentially damages CNS areas that experience extensive postnatal differentiation, such as the cerebellum and the dentate gyrus of the hippocampus, which results in the loss of granule cells in the dentate gyrus and Purkinje cells in the cerebellum, the thinning of cerebellar granule cell layers, and a decreased overall size and foliation of the cerebellum [9,24,25,26]. Transgenic expression of a BoDV-1 virulence factor, phosphoprotein (P), in mice causes neurobehavioral disturbances and CNS lesions, at least some of which were similar to those observed in persistently BoDV-1-infected rats [27,28]. These studies suggest that BoDV-1 infection causes neuropathology through neural circuit impairments. The generation of neuronal cells and the development of the connection between them are two major processes that may affect neural circuit formation; however, it is still unclear whether neuronal differentiation processes are disturbed by BoDV-1 infection and the molecular basis underlying neural circuit impairments.

In the BoDV-1-infected brain, upregulated expression of chemokine genes, such as *IP-10* and *RANTES*, and downregulated expression of neurotrophic factors, such as *BDNF* and *NGF*, have been observed [29,30,31,32,33]. The expression of BDNF and serotonin receptors is downregulated in P-transgenic (P-Tg) mice, similar to that in BoDV-1-infected rodents [27]. Additionally, neurotrophic insulin-like growth factor (IGF) signaling is dysregulated by the upregulation of the insulin-like growth factor binding protein 3 (IGFBP3) expression in P-Tg mice [28]. These transcriptomic changes impair CNS architecture and/or functions in P-Tg and BoDV-1-infected animals [28,30]. These observations clearly indicate that BoDV-1 infection alters the host transcriptome in the CNS, which causes BoDV-1-related neuropathology.

Here, we investigated the effects of BoDV-1 infection on neuronal differentiation and gene expression to reveal BoDV-1-related neuropathology. While BoDV-1 did not affect neuronal differentiation processes, it altered the gene expression profile of differentiated neuronal cells. Anti-BoDV-1 treatment attenuated the impairment of the expression of a part of dysregulated genes. Our findings suggest that anti-BoDV-1 treatment can improve some aspects of BoDV-1-related neuronal cell dysfunction and provide a molecular basis for the improvement of BoDV-1 neuropathology.

## 2. Materials and Methods

### 2.1. Cells

SH-SY5Y cells (a human neuroblastoma cell line; ATCC #CRL-2266) were cultured in Dulbecco’s Modified Eagle Medium (DMEM, Nakalai, Japan) containing Ham’s F12 supplemented with 10% fetal bovine serum (FBS, Sigma-Aldrich, St. Louis, MO, USA) and 1% non-essential amino acids (NEAA, Gibco, Grand Island, NY, USA). Persistently BoDV-1-infected SH-SY5Y (SH-SY5Y/BoDV) cells were established in a previous study [22]. Briefly, SH-SY5Y cells were infected with the huP2Br strain of BoDV-1 [34] that were prepared as cell lysate in DMEM supplemented with 2% FBS of OL cells persistently infected with the huP2Br strain of BoDV-1 [34]. BoDV-1-infected and mock (uninfected OL cell lysate)-infected cells were passaged for more than 2 months exactly in the same passage history, finally obtaining SH-SY5Y/BoDV and uninfected SH-SY5Y cells. SH-SY5Y cells were differentiated by treatment with 10 µM retinoic acid (RA) (Sigma-Aldrich, St. Louis, MO, USA), the stock solution of which was dissolved in DMSO, for two days, as described previously [35]. Morphological changes were evaluated using an ECLIPSE Ts2-FL microscope (Nikon, Tokyo, Japan). Neurites were defined as cell protrusions longer than soma. Total neurite length was measured for each cell. Neurites of more than 50 cells were evaluated for each condition. Cell viability was evaluated using a CellTiter-Glo Luminescent Cell Viability Assay (Promega, Madison, WI, USA) according to the manufacturer’s instructions. The luminescence of cell lysates was measured using a GloMax Discover microplate reader (Promega, Madison, WI, USA). Each luminescence was presented as a percentage of that of the scrambled siRNA-treated undifferentiated SH-SY5Y cells.

### 2.2. Real-Time RT-PCR

Real-time RT-PCR was conducted as previously described, with some modifications [22,36,37]. Briefly, total RNA was extracted from the indicated cells and reverse-transcribed using a Verso cDNA Synthesis Kit (Thermo Fisher Scientific, Waltham, MA, USA) according to the manufacturer’s instructions. Quantitative real-time RT-PCR (RT-qPCR) assays were performed using Fast SYBR Green Master Mix (Thermo Fisher Scientific, Waltham, MA, USA) and gene-specific primers with QuantStudio 6 (Thermo Fisher Scientific, Waltham, MA, USA). RT-qPCR assays for BoDV-1 X/P mRNA, M/G/L mRNA, and BoDV-1 gRNA were carried out using Luna Universal Probe qPCR Master Mix (New England Biolabs, Ipswich, MA, USA) and BoDV-1-specific primers and probes. Gene-specific primers and probes used in this study are listed in Table S1. We calculated the expression of each gene using the ∆∆Ct method. Thus, the expression was normalized with that of the *GAPDH* gene in each condition and the expression value was presented as a percentage of the control indicated in each graph.

### 2.3. RNA-Seq Analysis

Total RNA was isolated from RA-treated or DMSO-treated SH-SY5Y and RA-treated SH-SY5Y/BoDV cells. Library preparation from total RNA was performed using a TruSeq-stranded mRNA sample prep kit (Illumina, San Diego, CA, USA) according to the manufacturer’s instructions. Sequencing was performed on the Illumina NovaSeq 6000 platform. Sequenced reads were mapped to the human reference genome sequence (hg38) using HISAT2. Fragments per kilobase of exon per million mapped fragments (FPKMs) were calculated using Cufflinks. We set the cut-off FPKM value at 0.1 to remove lowly expressed transcripts. To select differentially expressed genes, we further set the cut-off FPKM value at 50 (average of all gene expression) for genes in RA-treated SH-SY5Y cells as we sought to extract the genes whose expression is upregulated in RA-treated SH-SY5Y cells compared with both DMSO-treated SH-SY5Y cells and RA-treated SH-SY5Y/BoDV cells. We defined “differentiation-related” genes as genes that were more than 2.5-fold upregulated in RA-treated SH-SY5Y cells compared with DMSO-treated control cells because the expression of a differentiation marker, *NCAM2*, is reportedly ~2-fold upregulated in RA-treated SH-SY5Y cells [38]. Genes that were more than 2.5-fold downregulated by BoDV-1 infection were identified by comparing the expression between RA-treated SH-SY5Y/BoDV cells and RA-treated control SH-SY5Y cells. Scatter plots of gene expression were generated using Python 3.9. We further selected differentiation-related genes that were downregulated by BoDV-1 infection. A heatmap of the expression of selected genes was generated using Python 3.9.

### 2.4. siRNA Transfection

Cells were transfected with the indicated siRNAs using Lipofectamine RNAiMAX (Invitrogen, Carlsbad, CA, USA) at a final concentration of 10 nM. The sequences of the siRNAs used in this study are as follows:

si-Control, MISSION Negative control (SIC001; Sigma-Aldrich, St. Louis, MO, USA)

si-BoDV N #2, 5′-UCU UAC UCC AGU AAA ACG CUG-3′ and 5′-GCG UUU UAC UGG AGU AAG AAG-3′ [22]

si-BoDV L #4, 5′-UCA AGU UGA AGC AGU UUU UUU-3′ and 5′-AAA AAC UGC UUC AAC UUG ACC-3′ [22]

TD-Borna, a cocktail of si-BoDV N #2 and si-BoDV L #4 [22]

### 2.5. Western Blot

Western blot was conducted as described previously [22]. Briefly, cell homogenates were subjected to SDS-PAGE and transferred onto polyvinylidene difluoride membranes. The membranes were then blocked with 5% skim milk and incubated with a mouse monoclonal antibody to BoDV-1 N (HN321) [37], BoDV-1 P (HP062) [37], or β-tubulin (Sigma-Aldrich, St. Louis, MO, USA). After three washes with 0.05% Tween 20 in TBS, the membranes were incubated with horseradish peroxidase-conjugated secondary antibodies for 1 h at 37 °C. The bound antibodies were detected using a Clarity Western ECL Substrate (Bio-Rad, CA, USA).

### 2.6. Statistics

Statistical significance was assessed using a two-tailed Student’s *t*-test with a threshold of *p* < 0.05.

## 3. Results

### 3.1. Impact of BoDV-1 Infection on Neuronal Differentiation in SH-SY5Y Cells

**Figure 1:**
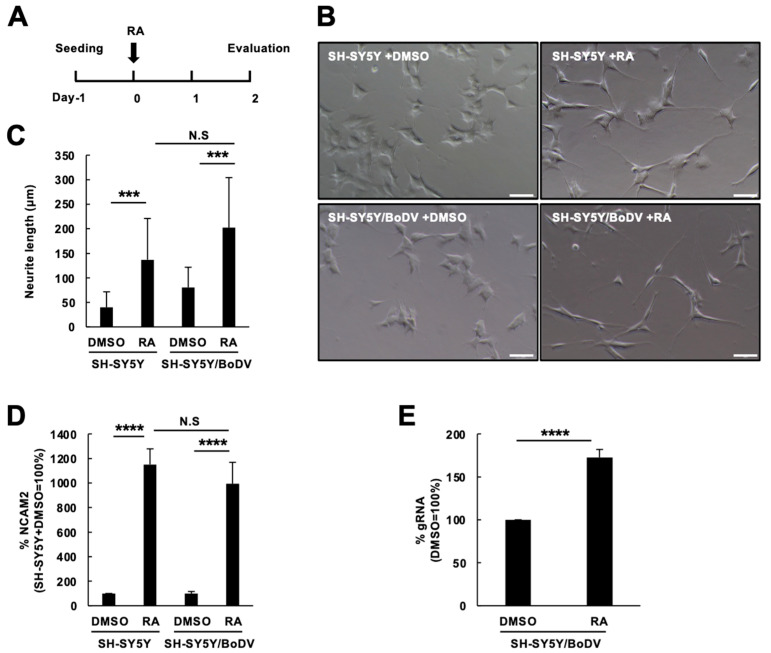
Neuronal differentiation of BoDV-1-infected SH-SY5Y cells. (**A**) Differentiation protocol of SH-SY5Y cells used in this study. SH-SY5Y cells were differentiated with 10 µM retinoic acid (RA) for 2 days. (**B**–**D**) Neuronal differentiation of BoDV-1-infected and uninfected SH-SY5Y cells. Neuronal differentiation was evaluated with the cell morphological changes (**B**), the total length of neurites in a cell (**C**), and the *NCAM2* gene expression (**D**). (**E**) BoDV-1 infection in differentiated (RA) and undifferentiated (DMSO) SH-SY5Y/BoDV cells. Bars, 30 µm. We calculated the expression of each gene using the ∆∆Ct method. The expression of each gene was normalized with that of the *GAPDH* gene in each condition. Values are expressed as the mean percentage + standard error of the mean (SEM) of at least three independent experiments. N.S, non-significant; ***, *p* < 0.005; ****, *p* < 0.001.

To reveal the impact of BoDV-1 infection on neuronal differentiation in more detail, we induced the differentiation of SH-SY5Y neuroblastoma cells using an established protocol [35]. Since retinoic acid (RA) was used in the protocol, we evaluated whether the expression of RA receptors was affected by BoDV-1 infection. The expression of the RA receptors α, β, and γ (RARA, RARB, and RARG, respectively) was not affected by BoDV-1 (Supplementary Figure S1). Although RA-induced differentiation of SH-SY5Y cells usually takes three to five days, the protocol used in this study successfully differentiated SH-SY5Y cells in two days, in agreement with a previous report [35], which was confirmed by extensive production of neurites and induction of neuronal markers in RA-treated cells (Figure 1). Therefore, we used this protocol and examined the morphological effect of BoDV-1 infection on RA-induced neuronal differentiation by measuring the sum of neurite length in each cell after treatment with 10 µM RA for 2 days. As shown in Figure 1B,C, RA treatment differentiated persistently BoDV-1-infected SH-SY5Y (SH-SY5Y/BoDV) cells comparable to uninfected SH-SY5Y cells. We also evaluated the BoDV-1 effect by measuring a neuronal marker, *NCAM2* [38], using real-time RT-qPCR. RA treatment induced NCAM2 expression in SH-SY5Y/BoDV cells comparable to that in SH-SY5Y cells (Figure 1D). As BoDV-1 is a highly neurotropic virus, we hypothesized that BoDV-1 replicates more efficiently in differentiated cells than in undifferentiated cells. Consistent with this hypothesis, the BoDV-1 load in RA-treated differentiated SH-SY5Y/BoDV cells was higher than that in undifferentiated cells (Figure 1E). These results suggest that BoDV-1 infection does not have a detectable impact on neuronal differentiation processes and that BoDV-1 replicates more efficiently in differentiated neuronal cells.

### 3.2. Impairment in the Induction of Differentiation-Related Genes by BoDV-1 Infection

**Figure 2:**
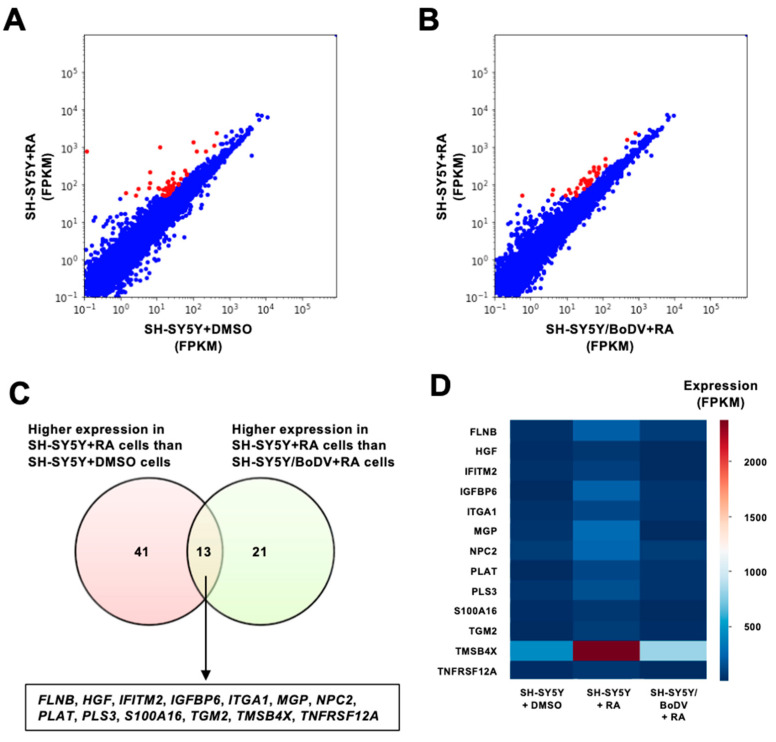
Differentiation-related genes affected by BoDV-1 infection. (**A**,**B**) Scatter plots of RNA-seq analysis. Extracted genes are indicated in red. (**A**) Comparison between undifferentiated (X-axis) and differentiated (Y-axis) SH-SY5Y cells. (**B**) Comparison between differentiated SH-SY5Y/BoDV (X-axis) and differentiated SH-SY5Y cells (Y-axis). (**C**) Venn diagram showing the number of differentiation-related (left circle) and BoDV-1-impaired (right circle) genes identified by RNA-seq. The overlapped genes are indicated. (**D**) Heatmap showing the expression levels of BoDV-1-impaired, differentiation-related genes.

Although BoDV-1 showed a minimal impact on neuronal differentiation processes apparently, it may affect the properties of differentiated cells at the transcriptome level. To reveal the impact of BoDV-1 on the transcriptome of differentiated cells, total RNA was extracted from undifferentiated (DMSO-treated) SH-SY5Y, differentiated (RA-treated) SH-SY5Y, or differentiated SH-SY5Y/BoDV cells and subjected to RNA-seq analyses. We compared the transcriptome of undifferentiated SH-SY5Y cells with that of differentiated SH-SY5Y cells and identified 54 differentiation-related genes that were upregulated in SH-SY5Y cells following RA treatment (Figure 2A,C). We also compared the transcriptome of differentiated SH-SY5Y cells with that of differentiated SH-SY5Y/BoDV cells and identified 34 genes that were downregulated by BoDV-1 infection (Figure 2B,C). Although the expression of three fourths of the differentiation-related genes was not impaired by BoDV-1 infection, we identified that one fourth was downregulated by BoDV-1 infection. These genes included *FLNB*, *HGF*, *IFITM2*, *IGFBP6*, *ITGA1*, *MGP*, *NPC2*, *PLAT*, *PLS3*, *S100A16*, *TGM2*, *TMSB4X*, and *TNFRSF12A* (Figure 2C,D). Among 13 genes, the expression changes of 12 genes were confirmed by real-time RT-qPCR analyses (Supplementary Figure S2). These results suggest that BoDV-1 affects the expression of a part of differentiation-related genes in differentiated neuronal cells.

### 3.3. Effect of a siRNA Cocktail, TD-Borna, on BoDV-1 Infection and the Expression of Differentiation-Related Genes Impaired by BoDV-1 Infection

**Figure 3:**
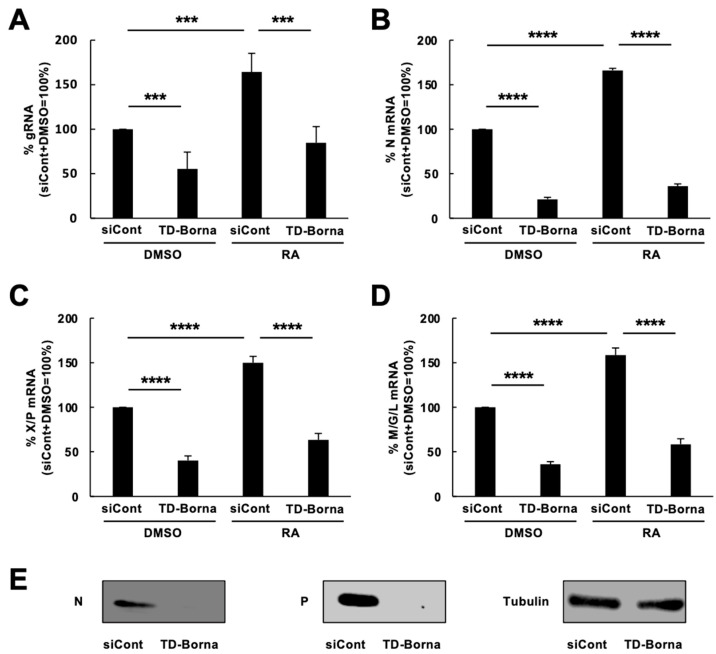
Suppression of BoDV-1 infection by TD-Borna treatment. (**A**) Effects of TD-Borna treatment on BoDV-1 load in SH-SY5Y/BoDV cells. SH-SY5Y/BoDV cells were treated with DMSO or RA in the presence or absence of TD-Borna for 2 days. The amounts of BoDV-1 gRNAs in SH-SY5Y/BoDV cells were determined by RT-qPCR analyses. (**B**–**D**) The amounts of N (**B**), X/P (**C**), and M/G/L (**D**) mRNAs in SH-SY5Y/BoDV cells. The amounts of BoDV-1 mRNAs were determined by RT-qPCR analyses. (**E**) The amounts of BoDV-1 proteins in SH-SY5Y/BoDV cells. The amounts of BoDV-1 N, BoDV-1 P, and tubulin were determined by western blot analyses using specific antibodies. siCont, the scrambled siRNA-treated control; TD-Borna, the siRNA cocktail against BoDV-1. We calculated the expression of each gene using the ∆∆Ct method. The expression of each gene was normalized with that of the *GAPDH* gene in each condition. Values are expressed as the mean percentage + SEM of four independent experiments. ***, *p* < 0.005; ****, *p* < 0.001.

**Figure 4:**
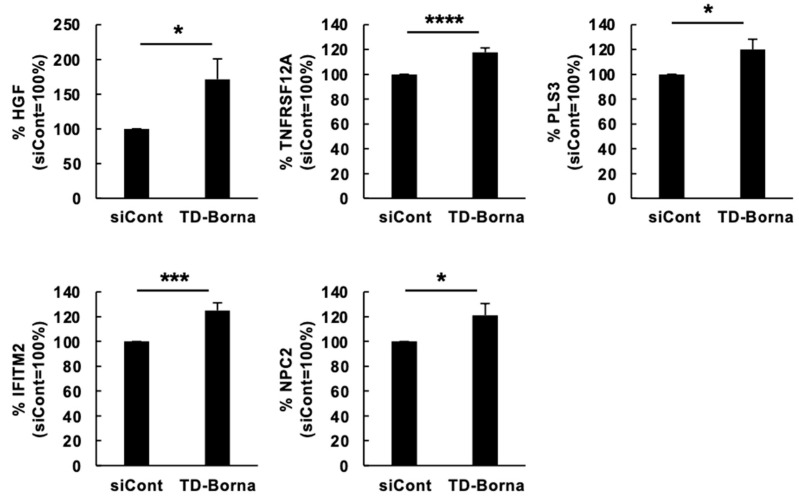
Effect of TD-Borna treatment on BoDV-1-related transcriptomic changes in differentiated SH-SY5Y cells. SH-SY5Y/BoDV cells were treated with RA in the presence of the scrambled siRNA (siCont) or TD-Borna for 2 days. The mRNA amounts of differentiation-related genes in the indicated cells were determined by RT-qPCR analyses. We calculated the expression of each gene using the ∆∆Ct method. The expression of each gene was normalized with that of the *GAPDH* gene in each condition. Values are expressed as the mean percentage + SEM of four independent experiments. *, *p* < 0.05; ***, *p* < 0.005; ****, *p* < 0.001.

We next investigated whether the transcriptome alterations induced by BoDV-1 infection were relieved by anti-BoDV-1 treatment. Previously, we developed TD-Borna, a cocktail of siRNAs against N and M/G/L mRNAs that has the potential to combat bornavirus infection in persistently bornavirus-infected cells [22]. TD-Borna is the only known bornavirus treatment that effectively reduces BoDV-1 load in SH-SY5Y/BoDV cells [22]. Therefore, we used TD-Borna to evaluate its effect on the expression of differentiation-related genes impaired by BoDV-1 infection. Consistent with a previous report [22], TD-Borna effectively reduced BoDV-1 load and three BoDV-1 mRNAs in differentiated SH-SY5Y/BoDV cells (Figure 3A–D). At the protein level, TD-Borna almost eliminated BoDV-1 proteins from infected cells (Figure 3E), suggesting that replication- and/or transcription-competent BoDV-1 RNPs are severely decreased by TD-Borna treatment. In this setting, TD-Borna treatment successfully relieved the BoDV-1-mediated impairment in the expression of several differentiation-related genes, such as *HGF*, *IFITM2*, *NPC2*, *PLS3*, and *TNFRSF12A* (Figure 4). In contrast, TD-Borna treatment did not exert any effect on the impaired expression of other differentiation-related genes, such as *FLNB*, *ITGA1*, *MGP*, and *TMSB4X* (Supplementary Figure S3). These results suggest that treatment of BoDV-1 infection can relieve some of the transcriptomic impairments induced by BoDV-1 infection, whereas some of the BoDV-1 transcriptomic changes remain after treatment.

### 3.4. Effect of TD-Borna on Cell Viability during Neuronal Differentiation in BoDV-1-Infected SH-SY5Y Cells

**Figure 5:**
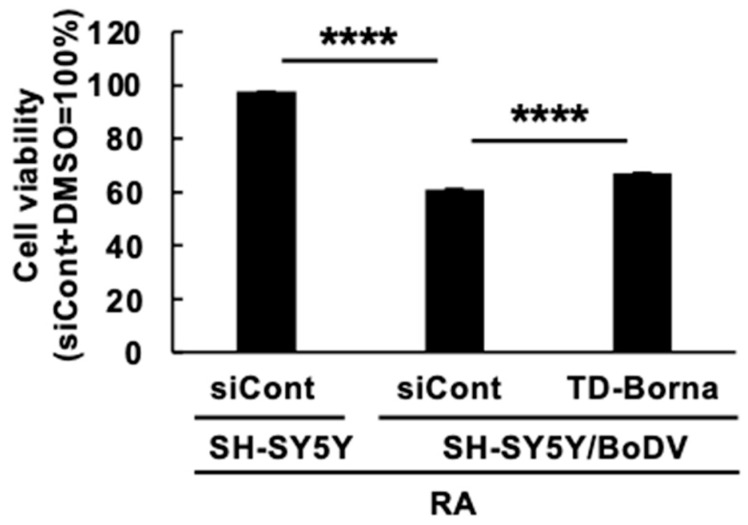
Effect of TD-Borna treatment on cell viability during differentiation of SH-SY5Y/BoDV cells. SH-SY5Y or SH-SY5Y/BoDV cells were treated with RA in the presence of the scrambled siRNA (siCont) or TD-Borna for 2 days. The cell viability was evaluated by using a CellTiter Glo Luminescent Cell Viability Assay. Values are expressed as the mean percentage + SEM of three independent experiments. ****, *p* < 0.001.

As all of the differentiation-related genes that respond to anti-BoDV-1 treatment, i.e., *HGF*, *IFITM2*, *NPC2*, *PLS3*, and *TNFRSF12A*, are involved in the apoptosis pathway, we finally evaluated cell viability during neuronal differentiation. RA-treated SH-SY5Y cells exhibited more than 90% of viability, whereas the viability of RA-treated SH-SY5Y/BoDV cells was ~60%, suggesting that BoDV-1 infection impairs cell viability during neuronal differentiation (Figure 5). TD-Borna slightly but significantly attenuated this impairment in cell viability. These results suggest that TD-Borna can relieve BoDV-1-mediated impairment in cell viability during neuronal differentiation.

## 4. Discussion

In this study, we obtained three novel insights into BoDV-1 infection in neuronal differentiation. First, BoDV-1 infection did not have a detectable impact on intracellular neuronal differentiation processes (Figure 1). Second, BoDV-1 impaired the expression of several differentiation-related genes in differentiated neuronal cells (Figure 2). Third, some BoDV-1-related transcriptomic changes and impairment in cell viability during neuronal differentiation were relieved by treatment of BoDV-1 infection (Figure 3, Figure 4 and Figure 5).

While we did not detect impairments in intracellular neuronal differentiation processes (Figure 1), BoDV-1 reportedly infects neural progenitor cells and inhibits neurogenesis, especially GABAergic neurogenesis [39,40,41,42]. Furthermore, neuronal differentiation of BoDV-1-infected PC12 cells in response to nerve growth factor (NGF) is also impaired [43]. These results suggest that BoDV-1 infection may impair the expression of soluble factors and/or receptors for soluble factors that trigger neuronal differentiation but not intracellular differentiation processes. Indeed, NGF receptors are downregulated in BoDV-1-infected PC12 cells [43], and transgenic expression of BoDV-1 P, a major virulence factor, in mice causes abnormal expression of neurotropic factors and neurotransmitters [27]. Alternatively, BoDV-1 infection does not impair all types of neurogenesis, and only a subpopulation of neurogenesis, for example GABAergic neurogenesis, might be disturbed by the infection. Further investigation is required to explain this discrepancy.

Although we demonstrated that BoDV-1 infection impaired the neuronal transcriptome (Figure 2 and Supplementary Figure S2), the underlying molecular mechanisms are unknown. BoDV-1 is a unique RNA virus that establishes a persistent infection in the cell nucleus [4,5,44]. During persistent infection, BoDV-1 closely associates with the host chromosomes to ensure intranuclear infection [45,46]. Since viral RNP binds to the host chromosome through its interaction with core histones and a host chromatin-remodeling DNA architectural protein, HMGB1 [45], BoDV-1 infection might affect host gene expression through these interactions. The expression of a chromatin-remodeling factor, MeCP2, is reportedly reduced in BoDV-1-infected neurons, suggesting that BoDV-1 infection might affect host gene expression through MeCP2 downregulation [47]. Reduced histone acetylation has been reported in BoDV-1 infection, which supports the theory that BoDV-1 infection affects host transcriptome through an epigenetic modification [30,48]. Furthermore, treatment with a histone deacetylase inhibitor attenuates the negative effects of BoDV-1 infection [30]. Based on these previous findings, we speculate that BoDV-1 infection may alter the neuronal transcriptome, possibly through modulations of host chromatin remodeling factors and/or epigenetic modifications such as histone acetylation.

Among 12 differentiation-related genes that were downregulated by BoDV-1 infection, five genes, i.e., *FLNB*, *HGF*, *ITGA1*, *PLS3*, and *TMSB4X*, are related to focal adhesion and cytoskeletal regulation, both of which are critical for neural circuit formation and synaptic function. For example, FLNB enhances cell motility via the phosphorylation of focal adhesion kinase [49]. PLS3 localizes to cell protrusions and plays crucial roles in cell motility through cross-linking actin filaments [50]. As BoDV-1 infection or BoDV-1 protein expression impairs neural circuit formation, synaptic plasticity, and memory [28,30,44,51], transcriptomic changes identified in this study might provide hints for uncovering molecular mechanisms of these BoDV-1-related impairments.

Among differentiation-related genes that were downregulated by BoDV-1 infection, anti-BoDV-1 treatment by TD-Borna could relieve the decrease in the expression of five genes, i.e., *HGF*, *IFITM2*, *NPC2*, *PLS3*, and *TNFRSF12A* (Figure 4). We noticed that all of these five genes are involved in apoptosis regulation: HGF, NPC2, and PLS3 are anti-apoptotic proteins, while IFITM2 and TNFRSF12A are pro-apoptotic proteins. HGF suppresses apoptotic signals through inhibition of caspase-3 activity, induction of anti-apoptotic proteins, such as Bcl-xL, or sequestration of Fas [52]. NPC2 is also shown to have anti-apoptotic effects [53]. IFITM2 induces apoptosis in a caspase-dependent, but p53-independent, manner [54]. PLS3 downregulation enhances P38 MAPK-mediated apoptosis [55]. BoDV-1 P is known to be phosphorylated by several kinases and acts as a decoy of the phosphorylation [56]. Taken together with our results, BoDV-1 infection may regulate PLS3-dependent apoptosis both transcriptionally and post-transcriptionally. TNFRSF12A is a receptor for tumor necrosis factor-like weak inducer of apoptosis (TWEAK), which activates multiple cell death pathways including apoptosis [57]. Although proteins encoded in the five above-mentioned genes contained both anti-apoptotic and pro-apoptotic proteins, we found that BoDV-1 infection decreased cell viability during neuronal differentiation and anti-BoDV-1 treatment by TD-Borna could attenuate this decrease (Figure 5). These results suggest that a decrease in the expression of anti-apoptotic genes plays more roles than that in the expression of pro-apoptotic genes in the BoDV-1-mediated decrease in cell viability during neuronal differentiation. Further investigation is required for the evaluation of this speculation.

In conclusion, although BoDV-1 does not have a detectable effect on intracellular differentiation processes, BoDV-1 infection affects the transcriptome in differentiated neuronal cells. As some of these BoDV-1 transcriptomic changes related to apoptosis were attenuated by antiviral treatment (Figure 4), it is important to treat BoDV-1 infection as early as possible to minimize BoDV-1-related neuronal cell dysfunction. Therefore, the development of additional therapeutic options will improve the outcome of BoDV-1 infection. Finally, because anti-BoDV-1 treatment did not relieve all BoDV-1 transcriptomic changes (Supplementary Figure S3), prevention of BoDV-1 infection is the best way to avoid BoDV-1-related neuronal cell dysfunction. In this sense, the development of a BoDV-1 vaccine is also an important issue for future investigations.

## Data Availability

The data of RNA-seq analysis were deposited in the GEO database (accession number GSE224811).

## Supplementary Materials

The following supporting information can be downloaded at: https://www.mdpi.com/article/10.3390/v15040942/s1, Figure S1: Comparable expression of receptors for RA in SH-SY5Y cells and SH-SY5Y/BoDV cells. Figure S2: Confirmation of RNA-seq results using real-time RT-qPCR analyses. Figure S3: Effect of TD-Borna on BoDV-1-related transcriptomic changes in differentiated SH-SY5Y/BoDV cells. Table S1. List of primers and probes used in this study.
