# Supplementary material for: Impact of Borna Disease Virus Infection on the Transcriptome of Differentiated Neuronal Cells and Its Modulation by Antiviral Treatment
Source: Viruses. 2023 Apr 10;15(4):942. doi: 10.3390/v15040942 (PMC10145824; doi:10.3390/v15040942)
Supplement: Supplementary file 1 [file viruses-15-00942-s001.zip › viruses-2248890-supplementary.pdf]

Table S1. List of primers and probes used in this study.

Primer	Sequence 5′to3′	Description
NCAM2-F	CTTATGTCTGCAGGGCCACA	Real-time RT-qPCR
NCAM2-R	TGCTGCCCTTTGACTTCGAT	Real-time RT-qPCR
RARA-F	CATTGAGACCCAGAGCAGCA	Real-time RT-qPCR
RARA-R	CCGGGTCACCTTGTTGATGA	Real-time RT-qPCR
RARB-F	GCACCAGGTATACCCCAGA	Real-time RT-qPCR
RARB-R	GTCGGTTCCTCAAGGTCCTG	Real-time RT-qPCR
RARG-F	CAAGGTGACCAGGAATCGCT	Real-time RT-qPCR
RARG-R	GTCTCCTGATGGGCTTTGCT	Real-time RT-qPCR
MGP-F	GCCGCCTTAGCGGTAGTAAC	Real-time RT-qPCR
MGP-R	AGCGTTCTCGGATCCTCTCT	Real-time RT-qPCR
HGF-F	ACCCTGGTGTTTCACAAGCA	Real-time RT-qPCR
HGF-R	GCAAGAATTTGTGCCGGTGT	Real-time RT-qPCR
S100A16-F	GCAGTCATTGTCCTGGTGGA	Real-time RT-qPCR
S100A16-R	GATGAGCTTATCCGCAGCCT	Real-time RT-qPCR
NPC2-F	CTGGCAGCTACATTCCTGCT	Real-time RT-qPCR
NPC2-R	CACGGCCTTGCTGCTTTTAG	Real-time RT-qPCR
ITGA1-F	GGCAGCACAATTCATGCACA	Real-time RT-qPCR
ITGA1-R	AAATGTACACAGCTCCCCCG	Real-time RT-qPCR
FLNB-F	GGAGTTGCTCGTGGAAGACA	Real-time RT-qPCR
FLNB-R	CCTGCAGCTTTGGTGTCAAC	Real-time RT-qPCR
TGM2-F	GGCGAACCACCTGAACAAAC	Real-time RT-qPCR
TGM2-R	CAGGTACTTGGTGCCACACT	Real-time RT-qPCR
IGFBP6-F	AACCGCAGAGACCAACAGAG	Real-time RT-qPCR
IGFBP6-R	TGGTCACAATTGGGCACGTA	Real-time RT-qPCR
PLAT-F	TACAGCCAGCCTCAGTTTCG	Real-time RT-qPCR
PLAT-R	TTCTGCCCAAGATCACCGTC	Real-time RT-qPCR
PLS3-F	ACTCTGCTTCTGCCATTGGG	Real-time RT-qPCR
PLS3-R	TCGGAGTAAAGCAGCCAAGG	Real-time RT-qPCR
**Primer**	**Sequence 5′to3′**	**Description**
TMSB4X-F	CTTCGCTTTTCCTCCGCAAC	Real-time RT-qPCR
TMSB4X-R	ATTTCACTGTCGTCCCACCC	Real-time RT-qPCR
IFITM2-F	AGCAGGAAGTGGCTATGCTG	Real-time RT-qPCR
IFITM2-R	CACGGAGTACGCGAATGCTA	Real-time RT-qPCR
TNFRSF12A-F	CGGACCTGGACAAGTGCAT	Real-time RT-qPCR
TNFRSF12A-R	GTCTCCTCTATGGGGGTGGT	Real-time RT-qPCR
GAPDH-F	AGCGAGATCCCTCCAAAATC	Real-time RT-qPCR
GAPDH-R	AAATGAGCCCCAGCCTTCTC	Real-time RT-qPCR
BoDV-1 PmRNA/gRNA probe	FAM-AGAACCCCTCCATGATCTCAGACCCAGA-TAMRA	Real-time RT-qPCR (Hayashi et al., 2009)
BoDV-1 gRNA-specific RT primer for huP2Br strain	TGTTGCGCTAACAACAAACCAATCAC	Real-time RT-qPCR (Hayashi et al., 2009)
BoDV-1 P mRNA/gRNA-forward primer for huP2Br strain	ATGCATTGACCCAACCAGTC	Real-time RT-qPCR
BoDV-1 P mRNA/gRNA-reverse primer for huP2Br strain	ATCATTCGACAGCTGCTCCCTTC	Real-time RT-qPCR
BoDV-1 N-forward primer (OU277)	GGAGCCGAGCAGATCAAGAA	Real-time RT-qPCR
BoDV-1 N-reverse primer (OU278)	CACAAAGGAGCCTACCCAGG	Real-time RT-qPCR
BoDV-1 L probe	FAM-CGAGGCATCCGTGGTCAGCAGAT-TAMRA	Real-time RT-qPCR
BoDV-1 L-forward	GGAAGCGCCCCGTGTT	Real-time RT-qPCR
BoDV-1 L-reverse primer (OU283)	CCCCCACAGTGATTCGCTTA	Real-time RT-qPCR

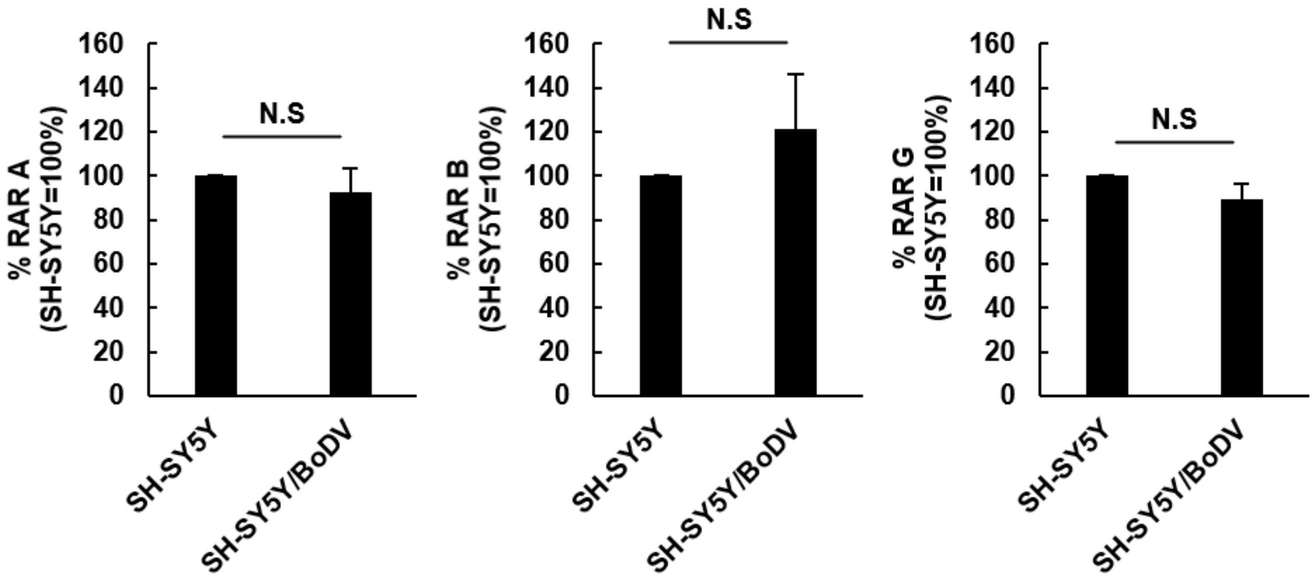

Figure S1. Comparable expression of receptors for RA in SH-SY5Y and SH-SY5Y/BoDV cells. The mRNA amounts of the RA receptors were evaluated by real-time RT-qPCR. We calculated the expression of each gene by the DDCt method. The expression of each gene was normalized with that of the *GAPDH* gene in each condition. Values are expressed as the mean percentage + standard error of mean (SEM) of three independent experiments. N.S, non-significant.

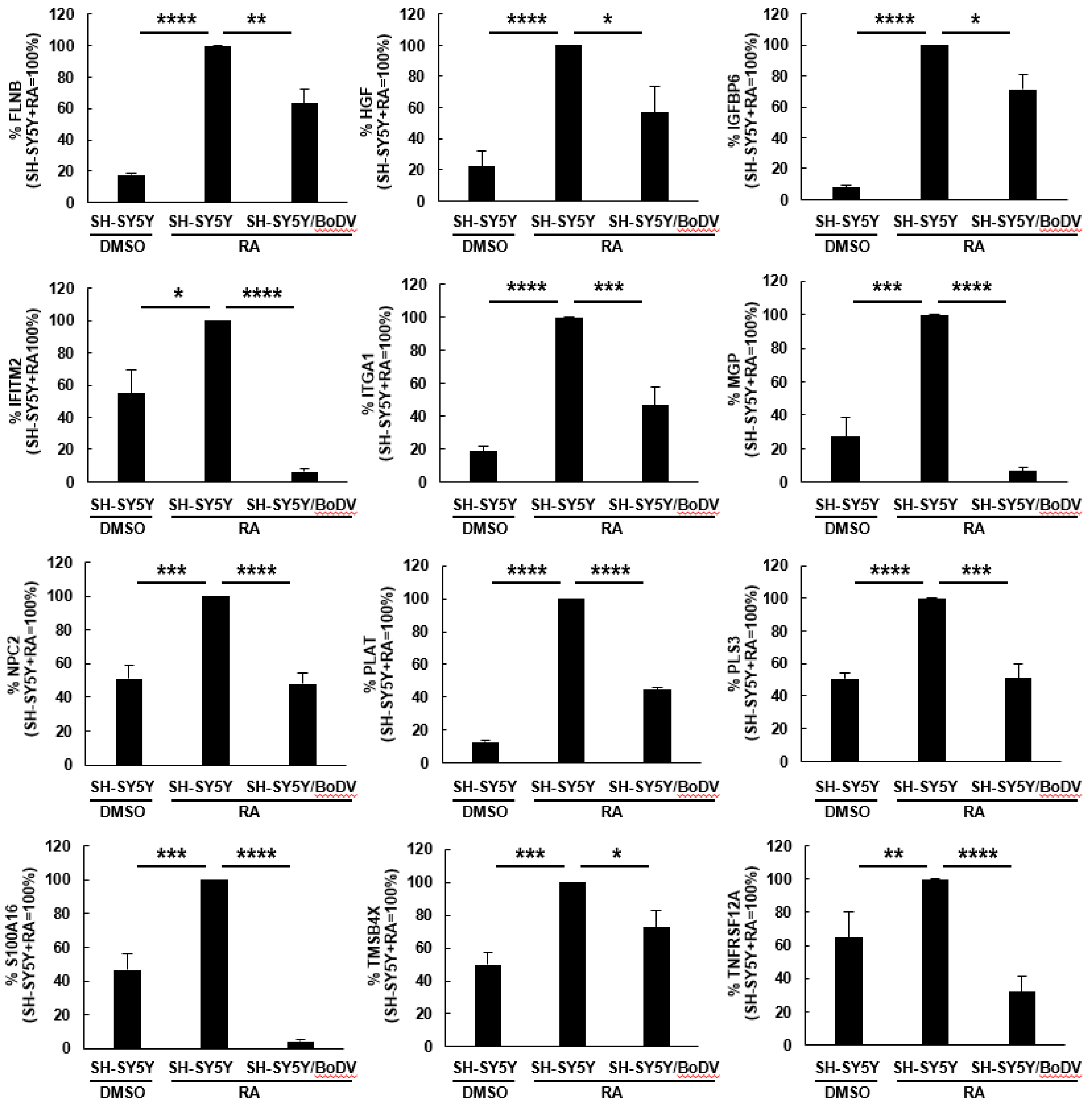

Figure S2. Confirmation of RNA-seq results using real-time RT-qPCR analyses. The mRNA amounts of differentiation-related genes that were downregulated by BoDV-1 infection in our RNA-seq analysis were evaluated by real-time RT-qPCR. We calculated the expression of each gene by the DDCt method. The expression of each gene was normalized with that of the *GAPDH* gene in each condition. Values are expressed as the mean percentage + SEM of at least three independent experiments. *, $P < 0.05$; **, $P < 0.01$; ***, $P < 0.005$; ****, $P < 0.001$.

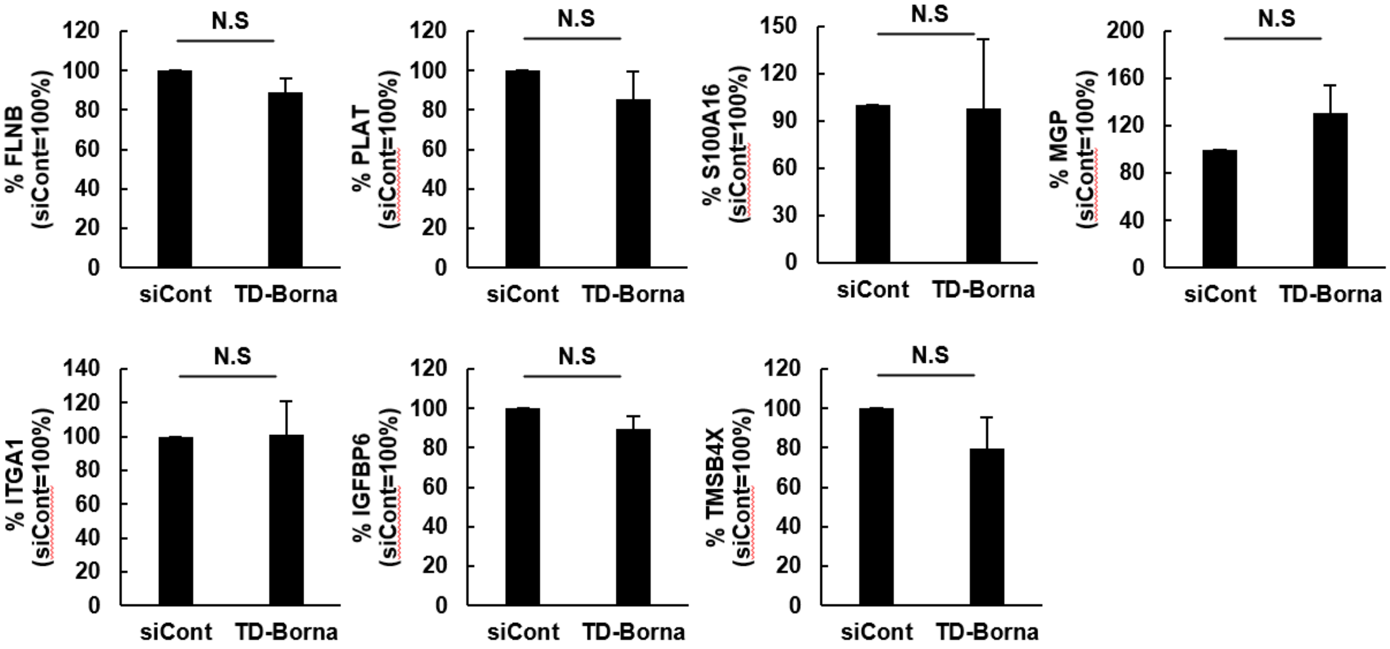

Figure S3. Effect of TD-Borna on BoDV-1-related transcriptomic changes in differentiated SH-SY5Y/BoDV cells. RA-treated SH-SY5Y/BoDV cells were treated with the scrambled siRNA (siCont) or TD-Borna. The mRNA amounts of differentiation-related genes that were downregulated by BoDV-1 infection were evaluated by real-time RT-qPCR. We calculated the expression of each gene by the DDCt method. The expression of each gene was normalized with that of the *GAPDH* gene in each condition. Values are expressed as the mean percentage + SEM of at least three independent experiments. N.S, non-significant.
